# Skeletal Class III Malocclusion with Lateral Open Bite and Facial Asymmetry Treated with Asymmetric Lower Molar Extraction and Lingual Appliance: A Case Report

**DOI:** 10.3390/ijerph18105381

**Published:** 2021-05-18

**Authors:** Vo Truong Nhu Ngoc, Nguyen Thi Thu Phuong, Nguyen Viet Anh

**Affiliations:** 1 School of Odonto Stomatology, Hanoi Medical University, Hanoi 100000, Vietnam; nhungoc@hmu.edu.vn (V.T.N.N.); drnguyenthuphuong70@gmail.com (N.T.T.P.); 2 Viet Anh Orthodontic Clinic, Hanoi 100000, Vietnam

**Keywords:** skeletal class III malocclusion, mandibular lateral deviation, lateral open bite, lingual orthodontics, molar extraction

## Abstract

A skeletal Class III malocclusion with open bite tendency is considered very difficult to treat orthodontically without surgery. This case report describes the lingual orthodontic treatment of an adult skeletal Class III patient with mandibular deviation to the left side, lateral open bite, unilateral posterior crossbite, zero overbite and negative overjet. The lower incisors were already retroclined to compensate with the skeletal discrepancy. The patient was treated by asymmetric molar extraction in the mandibular arch to retract the lower incisors and correct the dental midline, with the help of intermaxillary elastics. Lingual appliance was used with over-torqued lower anterior teeth’s brackets to control the torque of mandibular incisors. After a 30-month treatment, satisfactory smile and facial esthetics and good occlusion was achieved. A 12-month follow-up confirmed that the outcome was stable. Asymmetric molar extraction could be a viable option to retract mandibular incisors in Class III malocclusion with lower dental midline deviation.

## 1. Introduction

Nonsurgical treatment of skeletal Class III malocclusion associated with hyperdivergent facial pattern and open bite is very challenging [1,2]. The etiology of open bite and Class III malocclusion is multifactorial, including heredity and environmental factors [3,4,5,6]. Moreover, skeletal Class III treatment difficulty increases considerably when associated with facial asymmetry. The asymmetry usually includes mandible and dental midline shift, canted occlusal plane, unilateral crossbite and different posterior occlusion between the right and the left side [7,8]. Orthognathic surgery is a good option to correct the severe mandibular asymmetry with good result but patients may refuse surgery because of risks, high cost and long recovery time. Camouflage treatment of borderline asymmetry cases may involve asymmetric extraction or distalization, arch expansion or constriction, and cross elastic use, but these methods cannot treat severe cases and the outcome is sometimes compromised [9].

The extraction of premolars is commonly used to correct the anteroposterior discrepancy. If the third molars are present, extraction of the first or second molars could be a good alternative [10,11]. However, the disadvantage of molar extraction is long treatment time because the extraction space is larger. Moreover, preventing the tipping of molar into edentulous space requires additional orthodontic mechanics.

Since the first introduction in 1979, lingual appliances have become in demand for adult patients because of their invisibility [12,13]. Lingual tipping or loss of torque control of the incisors during space closing stage is observed more frequently in lingual orthodontics [14,15]. Preserving the torque of lower incisors during retraction is a critical factor in lingual orthodontic treatment of skeletal Class III patients.

This case report presents the lingual orthodontic treatment of an adult skeletal Class III patient with mandibular deviation to the left side, lateral open bite, unilateral posterior crossbite, zero overbite and negative overjet without orthognathic surgery. Lingual appliances were used along with asymmetric molar extraction and intermaxillary elastic to correct the anteroposterior and transverse problems. The smile esthetic was not affected during treatment thanks to invisible lingual brackets and good torque control of mandibular incisors was achieved.

## 2. Materials and Methods

### 2.1. Diagnosis and Etiology

**Figure 1:**
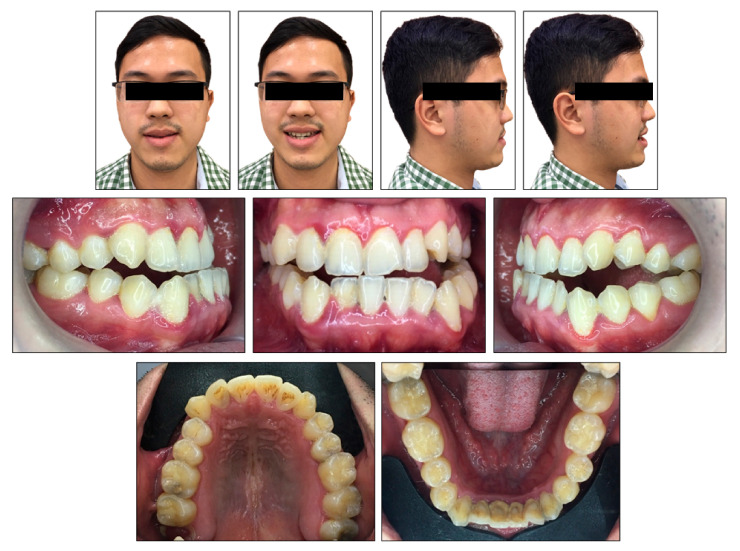
Pretreatment photographs.

The patient was a 24-year-old man with the chief complaint of lateral open bite, edge-to-edge incisor bite and lower dental midline deviation to the left side. He strongly desired an invisible orthodontic appliance for esthetic reasons. Extraorally, he had a concave profile with slightly prominent lower lip and chin. The mandible shifted to the left side and the upper occlusal plane was canted (Figure 1). No symptom of temporomandibular disorders was detected. The patient exhibited a habit of low position of the tongue at rest without tongue-thrust during swallowing.

**Figure 2:**
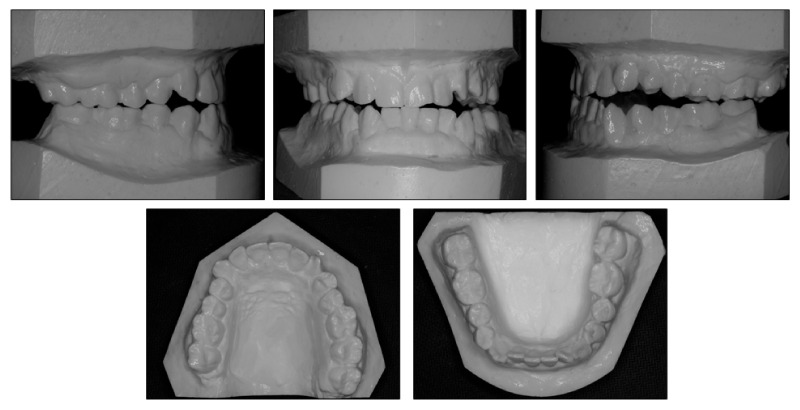
Pretreatment models.

Intraorally, the patient had Class III molar and canine relationship on the right side and Class I molar and canine relationship on the left side (Figure 2). The overbite was almost zero and the overjet was −0.8 mm. There was lateral openbite on both sides, which was more severe on the left side including the canines and premolars. The mandibular left canine, premolars and first molar were in crossbite. The upper dental midline was deviated 1 mm to the right side and the lower dental midline shifted 4 mm to the left side. There was mild crowding in both arches.

**Figure 3:**
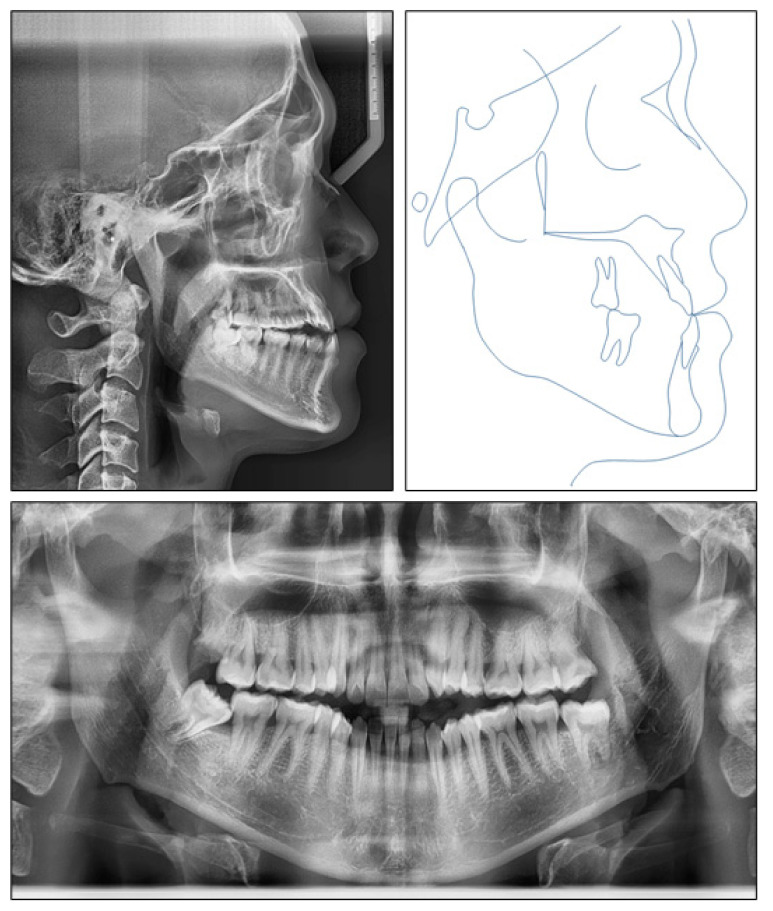
Pretreatment radiographs and tracing.

The panoramic radiograph indicated that four third molars were present, upper right third molar was hopeless and lower right third molar was mesially tipped. The lateral cephalometric analysis showed a skeletal Class III jaw relationship with mandibular protrusion (ANB, −2.1°; Wits appraisal, −8.1 mm; SNB, 87°), and hyperdivergent facial pattern (FMA, 29°). The dental compensation could be seen with upper incisor proclination (U1-SN, 112°) and lower incisor retroclination (L1-MP, 77.2°). Both the upper and lower lips were behind the E-line (Figure 3).

### 2.2. Treatment Objectives

The following treatment objectives were established: (1) eliminate lateral open bite on both sides and crossbite on the left side, (2) achieve normal overjet and overbite, (3) correct upper and lower midline deviation, (4) obtain Class I molar and canine on both sides, (5) correct the habit of low posture of the tongue at rest and (6) retract lower lips.

### 2.3. Treatment Alternatives

According to the treatment objectives, five treatment alternatives were proposed:

1. Orthognathic surgery would be used to set back the mandible and LeFort I osteotomy would correct the occlusal canting and mandibular lateral deviation. However, the patient refused surgery because of accompanying risks and costs;
2. Extraction of upper second premolars and lower first premolars would be performed to achieve normal overjet and overbite, obtain molar Class I relationship and correct midline deviation. However, extraction in the upper arch would retract the upper lip and make the profile more concave;
3. Extraction of upper third molars, lower right first premolar and left second premolar could be done. This option would not retract the upper lip, but the molar Class I relationship could not be achieved and the maxillary right second molar’s antagonist would be the mesially tipped third molar;
4. Extraction of upper third molars, lower right first molar and left second molar would be performed. With this approach, the upper lip would not be retracted, and molar Class I relationship could be achieved. The rationale of asymmetric molar extraction was that mandibular right first molar extraction would provide more strong posterior molar anchorage to correct midline deviation and canine Class III relationship on the right side;
5. Extraction of all four third molars would be applied in order to retract the entire lower arch with the help of temporary anchorage devices. However, the patient did not want to use any kind of skeletal anchorage. Additionally, first/second molar extraction would provide more space for correction of anteroposterior discrepancy.

### 2.4. Treatment Plan

Considering the patient’s requirement, the camouflage orthodontic treatment approach with asymmetric lower molar extraction without orthognathic surgery was chosen (option 4). The patient was informed that his concave profile and mandibular lateral deviation would not be corrected with this option.

### 2.5. Treatment Progress

All first molars and mandibular left third molar were banded, and the remaining teeth were bonded with a 0.018 × 0.018 in pre-adjusted edgewise multi-slot lingual appliance (CLB brackets, Hubit, Anyang-si, Gyeonggi-do, Korea) using indirect bonding in both arches. The lower incisor and canine bracket were 10° over torqued (55°). The teeth were aligned and leveled by using a sequence of round and square nickel-titanium continuous archwires of (0.012, 0.014, 0.016, 0.016 × 0.016 and 0.018 × 0.018 in). After one month, the mandibular right first molar and left second molar were extracted under local anesthesia.

**Figure 4:**
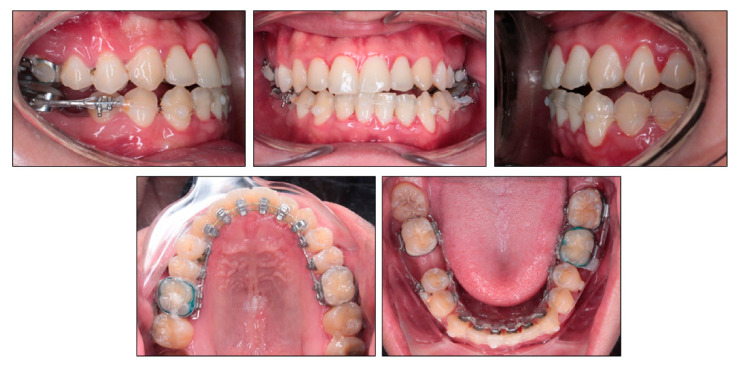
Space closure with labial and lingual elastic chains in the lower arch.

After seven months of treatment, the upper archwire was 0.018 × 0.018 in nickel titanium archwire and the lower archwire was 0.018 × 0.018 in stainless steel archwire. The space closure stage was initiated with elastic chains from mandibular right second molar to mandibular left third molar both lingually and labially to prevent rotation and transverse bowing effect (Figure 4). The right third molar tube was banded labially for easier handling. Clear composite buttons were bonded on the labial surface of mandibular canines for applying elastic chains. The labial bracket was bonded on the lower right second premolar and segmental archwire was placed between mandibular right second premolar and second molar to avoid tipping of these teeth into extraction space. On the initial space closure stage, the mandibular right third molar was not banded yet to allow spontaneous mesializing.

**Figure 5:**
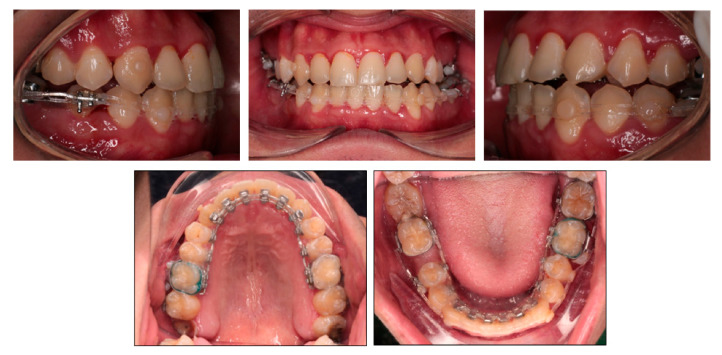
15-month treatment follow-up photographs.

After 15 months of treatment, the positive overbite and overjet were obtained (Figure 5). The lateral openbite on both sides was almost corrected with only 1 mm of open bite remaining. The lower midline deviation was significantly improved. The mandibular left second molar extraction space was completely closed, and the mandibular right first molar extraction space was closed by two thirds. A V-bend was applied to the labial segmental archwire in the lower right quadrant in order to bring upright the mandibular right second molar. The buccal crossbite on the left side became edge-to-edge bite. On the right side, the transverse buccal interdigitation was still not normal with lingually placed mandibular premolars and buccally placed maxillary premolars. Canine relationship was Class II in the left side.

**Figure 6:**
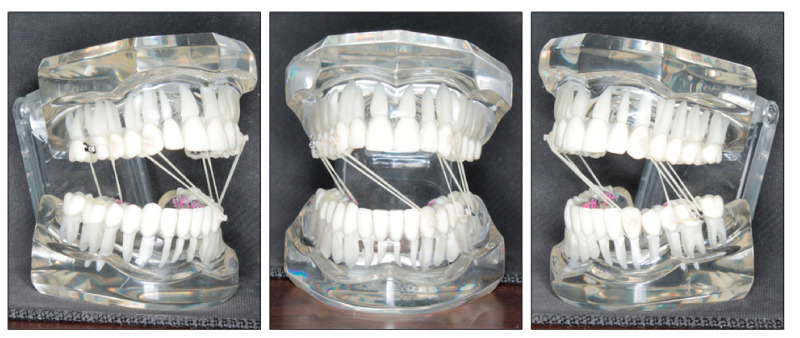
Applying intermaxillary elastics to correct transverse and sagittal discrepancies.

Over the next month, clear brackets (OK resin bracket, Hubit, Anyang-si, Gyeonggi-do, Korea) were bonded to the labial surface of maxillary premolars and segmental archwires were placed from maxillary first premolars to maxillary second molars in order to reinforce the maxillary buccal segment during the wearing of intermaxillary elastics. The cross elastics were applied from the lingual side of maxillary right second premolar to the labial side of mandibular right second premolar and first molar, from the lingual side of mandibular right first molar to the labial side of maxillary right second premolar and first molar and from mandibular left canine to maxillary right canine (Figure 6). Class II elastic was applied on the left side to correct the Class II canine relationship on that side.

**Figure 7:**
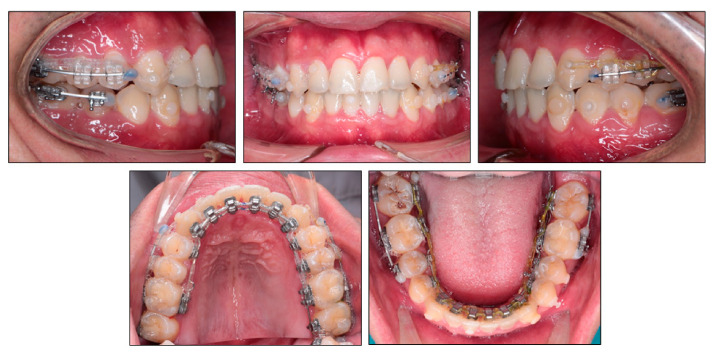
26-month treatment follow-up photographs.

After 26 months of treatment, all extraction spaces were completely closed (Figure 7). In the final space closing stage, molar bands were replaced by brackets to close the remaining band spaces and the mandibular right third molar was bonded to correct its mesial inclination. The dental midline was coincident and the transverse buccal interdigitation was almost normal with buccal cusps of maxillary premolars positioned buccal to buccal cusps of mandibular premolars. Final detailing of occlusion was obtained with 0.018 × 0.018 in nickel titanium archwire in both arches.

## 3. Results

**Figure 8:**
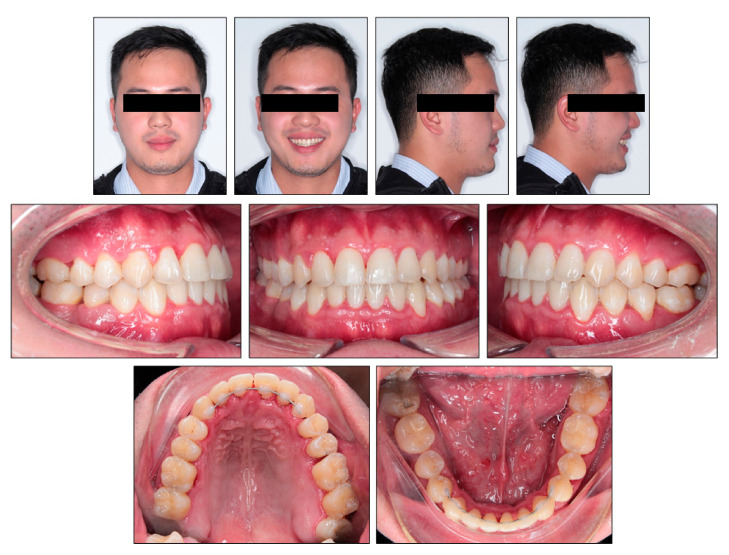
Posttreatment photographs.

**Figure 9:**
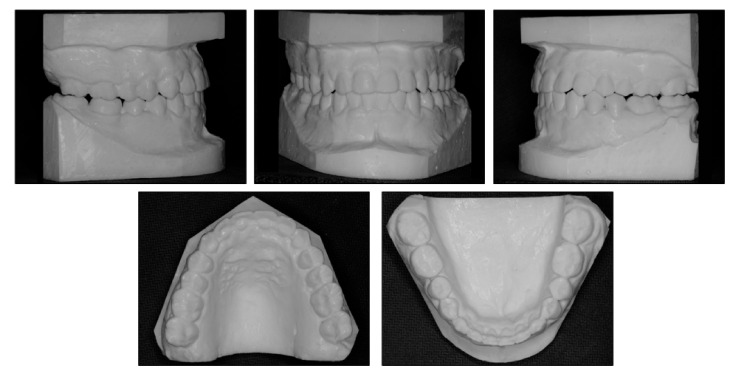
Posttreatment models.

**Figure 10:**
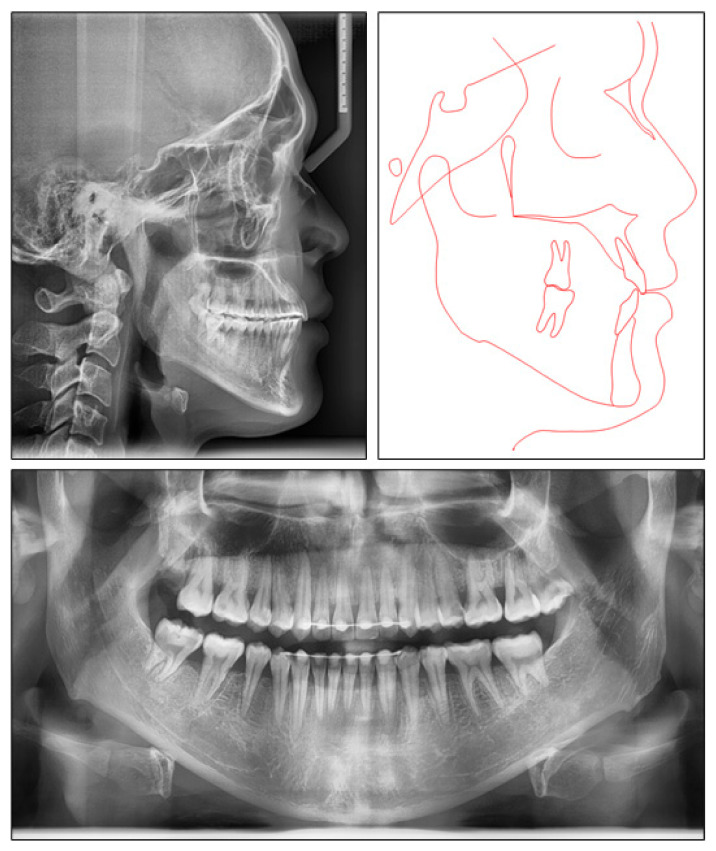
Post treatment radiographs and tracing.

**Figure 11:**
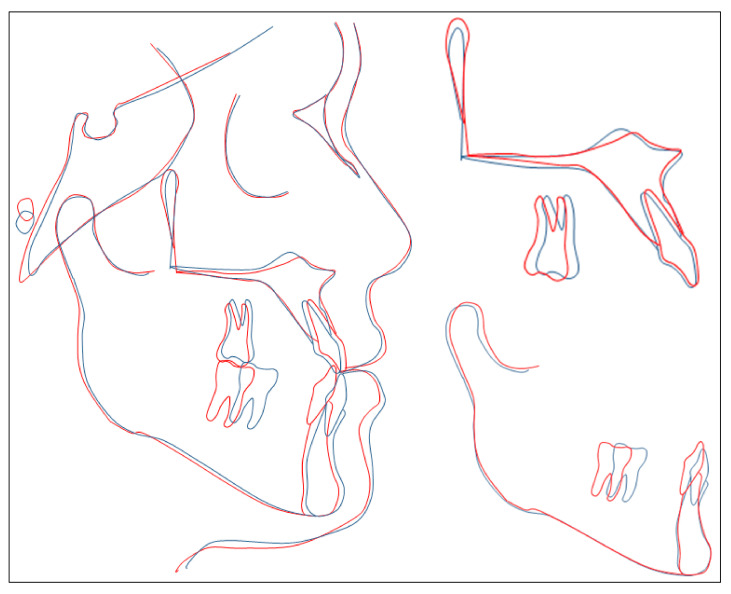
Overall and regional cephalometric superimpositions: blue, pretreatment; red, posttreatment.

**Figure 12:**
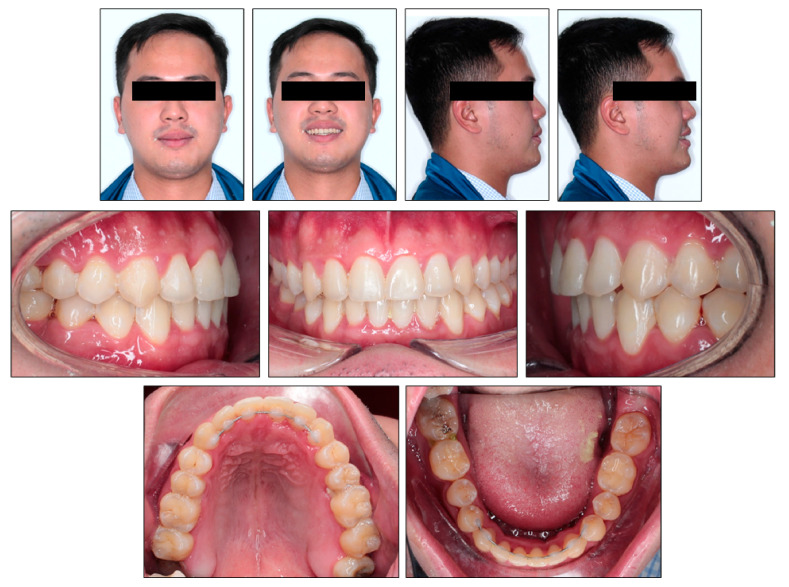
One-year retention photographs.

Evaluation of the treatment outcome showed that all treatment objectives were achieved with a good occlusion and improved facial esthetics (Figure 8, Figure 9 and Figure 10). The maxillary and mandibular arches were well aligned and leveled. All molar extraction spaces were closed with good interproximal contacts. Overjet and overbite were normalized. Class I canine and molar relationship was obtained on both sides (Figure 9). The good intercuspation was achieved without lateral open bite nor posterior crossbite. Both upper and lower dental midline were aligned with facial midline. The smile esthetic is considerably improved, despite persisting facial asymmetry including occlusal canting and mandibular deviation. The soft tissue profile was improved with retracted lower lips. The habit of low posture of the tongue at rest was corrected. The panoramic radiograph showed good root parallelism with minimal root resorption, the mesially tipped mandibular right third molar was made upright (Figure 10). The cephalometric analysis confirmed the improvement of skeletal Class III relationship (ANB, 0°; Wits appraisal, −0.3 mm) and good torque control of lower incisors during retraction (L1MP, 89.6°) (Table 1). These changes were further confirmed with cephalometric superimposition, which showed the bodily retraction of mandibular incisors, the maintenance of upper lip position and retraction of lower lip (Figure 11). The overall active treatment time was 30 months. After debonding of lingual appliance, permanent retainers were bonded in both arches to maintain long-term stability, along with clear retainers for night-time wearing. Photographs taken 1 year after treatment showed the result to be stable with only minimal relapse of lower dental midline deviation (Figure 12).

## 4. Discussion

Treatment of skeletal Class III malocclusion associated with open bite and posterior crossbite is very challenging [1]. Class III patients with hypodivergent facial pattern are easier to be treated nonsurgically because when the molars are extruded, the facial height increases and simultaneously the mandible displaces posteriorly, thus the Class III relationship is improved. However, in Class III patients with open bite tendency, the molar cannot be extruded because it makes vertical discrepancy worse. Moreover, posttreatment relapse is common in open bite cases regardless of surgical and nonsurgical treatment [16,17,18]. In these cases, anteroposterior movement of teeth is the main element to correct dental relationship, camouflage the skeletal discrepancy and improve profile.

In adult skeletal Class III patients, upper incisors are usually not moved forward because they are initially proclined. This creates excessive proclination of upper incisors, which is unesthetic, especially in Asian patients [19]. Therefore, the treatment approach of choice usually involves retraction of lower incisors. In mild to moderate cases, total mandibular arch distalization with skeletal anchorage could be applied [20,21,22]. In more severe Class III cases, extraction of four premolars could be done with caution because it may displace the upper lip backward and make the profile more concave [23]. Extraction of two mandibular premolars could only be a viable option when the lower incisors need to be retracted significantly, but this approach results in full-cusp Class III molar relationship. Mandibular molar extraction is also a good treatment approach because it creates enough space for correction of anteroposterior discrepancies. Moreover, a Class I molar relationship could be obtained after treatment, and the posttreatment dentition is similar to a nonextraction case without losing any premolars.

Mandibular incisors are usually retroclined to compensate with skeletal disharmony in skeletal Class III patients. Therefore, torque control of mandibular incisors during retraction is critical in order to keep them upright and not more retroclined. During closing extraction space in lingual orthodontics, lingual tipping of anterior teeth is more common than in labial orthodontics [14]. In this case report, the mandibular first molar extraction created a very large space, so it was even more challenging to control the torque of lower incisors. We used an over torqued bracket for mandibular incisors and canines with the torque of 55° (the regular torque of lower incisor bracket is 45°), combined with full size archwire (0.018 × 0.018 in stainless steel) to retract the lower anterior teeth bodily and prevent them from lingual tipping. A good result was obtained, in which the retroclination of mandibular incisors was improved (pretreatment L1-MP, 77.2; posttreatment L1-MP, 89.6) and root movement was achieved. Mandibular incisor retraction with good torque control may be the most important factor to make favorable change in the patient’s profile because root movement might create some alveolar bone remodeling and result in lower lip retraction.

As the molar extraction space is large, the adjacent teeth tend to tip into the extraction space. This happens because the archwire stiffness reduces when the interbracket span increases and the archwire cannot maintain a straight state. To overcome this problem, a V-bend could be applied to the archwire at the extraction space to bring the roots of adjacent teeth together. In the final stage of space closure when the interbracket span is short, the V-bend has to be removed to facilitate complete space closing.

In asymmetry cases, the asymmetry may have anteroposterior, transverse and vertical components. The orthodontists have to determine which components contribute to the asymmetry to plan suitable a treatment approach [24,25]. In this case report, the asymmetry has all three components: the anteroposterior component is the different molar and canine relationship between the right and left sides; the transverse component expresses as posterior crossbite on the left side; and the vertical component is the occlusal canting. The occlusal canting is very difficult to correct with a nonsurgical treatment approach, especially when it is associated with the lateral mandibular deviation, so we ignored the vertical asymmetric component in this case. The anteroposterior asymmetric component was treated with asymmetric mandibular molar extraction and asymmetric Class II elastics to correct the Class III molar relationship on the right side and move the lower dental midline to the right side. Despite of the initial canine and molar Class I on the left side, the rationale of extracting mandibular left second molar is to retract mandibular incisors and use anchorage from mandibular left third molar to move the upper dental midline to the left with Class II elastics. The transverse asymmetric component was corrected with cross elastics and anterior diagonal elastic. Special caution should be taken when using asymmetric intermaxillary elastics to avoid creating or aggravating occlusal canting [26]. Additionally, intermaxillary elastic requires great patient cooperation to be effective [27]. In this case report, the importance of wearing intermaxillary elastic was emphasized in each appointment so a good result was achieved.

Lingual appliances offer a special contribution of open bite correction due to patient’s myofunctional adaptation during treatment [28]. The stability of open bite treatment may be greatly increased if the associated abnormal oral functions are eliminated, particularly the low anterior tongue posture at rest and the tongue thrust during swallowing. The lingual brackets have spur effects, which modifies the tongue posture to a backward and upward position. Immediately after bonding, the tongue may be irritated by lingual appliances but later there is usually no irritation and bracket indentation on the tongue because a new tongue posture is established. This effect could be further reinforced with myofunctional and speech therapy.

## 5. Conclusions

A skeletal Class III malocclusion associated with lateral open bite, posterior crossbite and midline discrepancy was successfully treated with asymmetric mandibular molar extraction, intermaxillary elastics and lingual appliances. The treatment result remained stable at the 12-month follow-up examination.

Asymmetric molar extraction could be a viable option to retract mandibular incisors in Class III malocclusion with lower dental midline deviation.

## Figures and Tables

**Table 1:** Cephalometric skeletal analysis.

	Pretreatment	Posttreatment
SNA (°)	85.1	83.8
SNB (°)	87.2	83.8
ANB (°)	−2.1	0
Wits appraisal	−8.1	−0.3
FMA (°)	29.0	29.7
U1-SN (°)	110.9	107.0
U1-NA (°)	25.7	23.3
U1-NA (mm)	5.1	4.8
L1-MP (°)	77.2	89.6
L1-NB (°)	14.8	24.21
L1-NB (mm)	3.0	3.0
U1-L1 (°)	141.6	132.0
E-line-upper lip (mm)	−3.8	−2.8
E-line-lower lip (mm)	−0.4	−2.1

ANB, A point, nasion, B point; FMA, Frankfort mandibular plane angle; L1, lower central incisor; MP, mandibular plane; NA, nasion point A; NB, nasion point B; SNA, sella nasion point A; SNB, sella nasion point B; U1, upper central incisor.

## Data Availability

Not applicable.
